# Posttraumatic stress disorder neurophysiology and clinical correlates in pediatric critical care: conceptualizing a PICU-PTSD framework

**DOI:** 10.3389/fped.2025.1558302

**Published:** 2025-07-24

**Authors:** Rebecca E. Hay

**Affiliations:** ^1^ Nuffield Department of Primary Health Care Sciences, University of Oxford, Oxford, United Kingdom; ^2^ Paediatric Intensive Care, Birmingham Children's Hospital, Birmingham, United Kingdom

**Keywords:** pediatric critical care, PICU outcomes, posttraumatic stress disorder, neurocritical care, stress, pathophysiology, biopsychosocial model

## Abstract

Posttraumatic stress disorder (PTSD) is common in child and parent survivors of critical illness, with significant negative impact on life after survival. Understanding the neuroscience and pathophysiology of contributing factors to PTSD within the pediatric intensive care unit (PICU) context can help identify potentially modifiable risk factors, aid risk stratification, and identify knowledge gaps for further study. This narrative review explores the evidence-based neurophysiology of PICU-PTSD, summarizing predisposing and protective factors related to critical care and conceptualizing the disorder in a biopsychosocial framework.

## 1 Introduction

Pediatric intensive care unit (PICU) therapy or critical illness inherently involves exposure to actual or threatened death and critical injury, increasing the risk of post-traumatic stress disorder (PTSD) (1, 2). This is reflected in prevalence data, as found in our 2025 meta-analysis where as many as one in three children (29%) screened positive for PTSD six months after PICU admission (1). PTSD is a neuropsychiatric disorder characterized by functional, structural and pharmacological changes, following exposure to traumatic events (3). Exposure to overwhelming stressors like critical illness during early life and adolescence can negatively influence neurodevelopment across a lifespan (4, 5), and PTSD after PICU admission follows a more chronic disease trajectory when compared to other causes of PTSD (1, 6).

**Figure 1:**
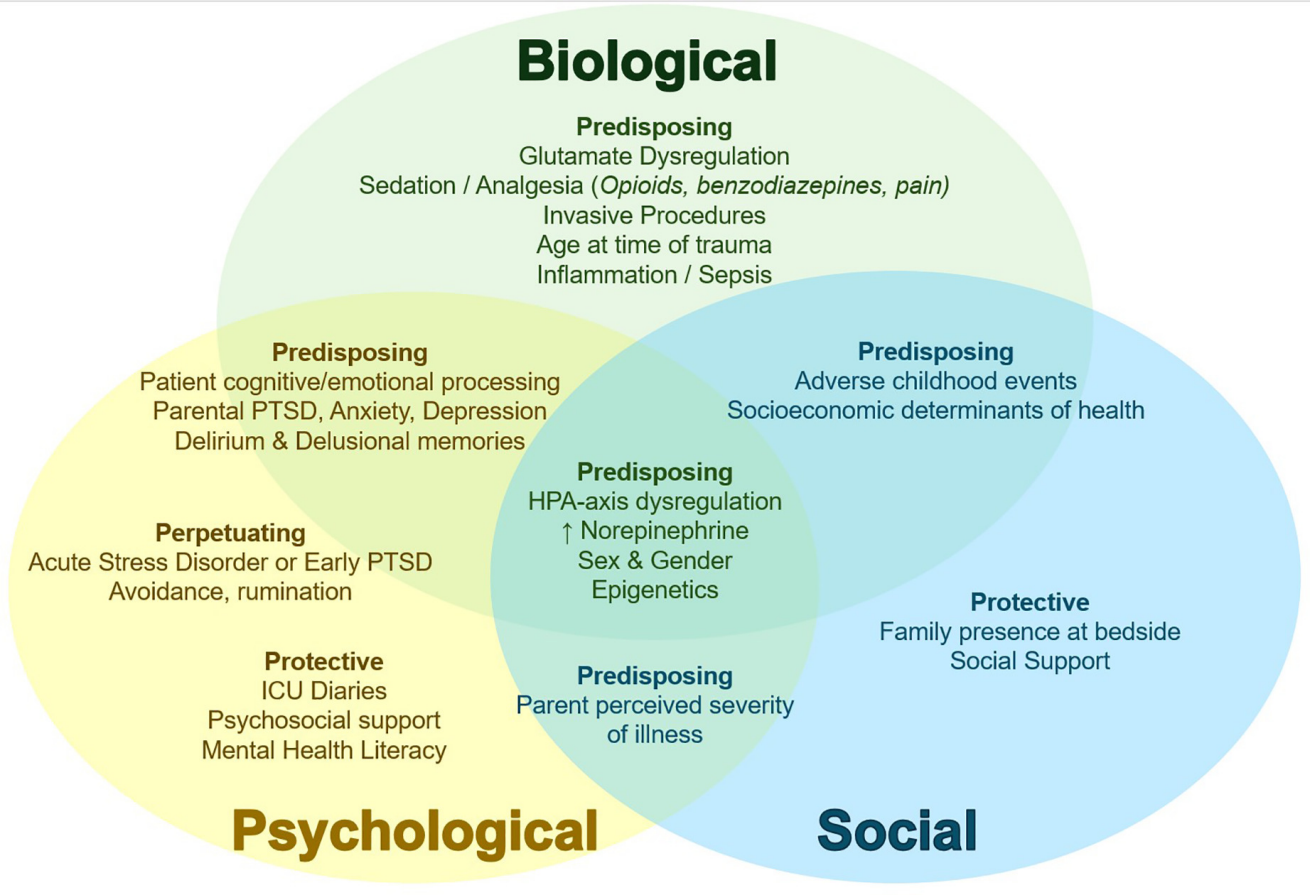
Conceptual biopsychosocial framework of PICU-PTSD. Biological (green text), psychological (yellow text), and social (blue text) contributors to PICU-PTSD depicted with relevant overlap in neighboring domains. Subheadings describe Predisposing factors (vulnerabilities for developing PTSD; some may be PICU-specific and potentially modifiable); Perpetuating factors (those that impede or limit recovery, or prolong PTSD course); and Protective factors (factors that reduce PSTD risk or augment recovery). ↑, increased. PICU, pediatric intensive care unit; PTSD, posttraumatic stress disorder; HPA, hypothalamic-pituitary-adrenal.

Understanding PTSD pathophysiology in the PICU context may give important clues on prevention and intervention. Here, we review suspected contributing factors and underlying neurophysiology associated with PTSD after PICU admission (PICU-PTD) (2, 7) and frame this knowledge as a biopsychosocial model (Figure 1). Biopsychosocial models provide a more comprehensive understanding of disease from traditional biomedical models, by recognizing and examining the impact of the individual whole (psychology) and environment (social context) on biology and health (8). Biological, psychological and social factors are inextricably linked and context dependent (8–10). Regarding PTSD, the unique pathogenesis of trauma-related disorders involving psychopathological and social dysfunction following traumatic stress in vulnerable individuals are well-described by biopsychosocial models (11, 12). Importantly, the framework proposed here is not meant to be a prescriptive separation of contributing factors (Overlap between biological/psychological/social visually demonstrated in Figure 1) and is not meant to be applied independent of individual context (9). In individual cases, this knowledge may be further framed according to subjective individual context, clinical relationships, and medical complexity (9), perhaps through person-centered dispositional models that consider individual weighting of contributing and protective factors (10).

## 2 Biological factors

Biological factors related to PICU-PTSD (Figure 1), particularly the complex interplay between the stress response, neurological circuitry and processing, and inflammation (3) are summarized in this section.

### 2.1 Overview of neuroendocrine response and fear neurocircuitry in pediatric PTSD

**Figure 2:**
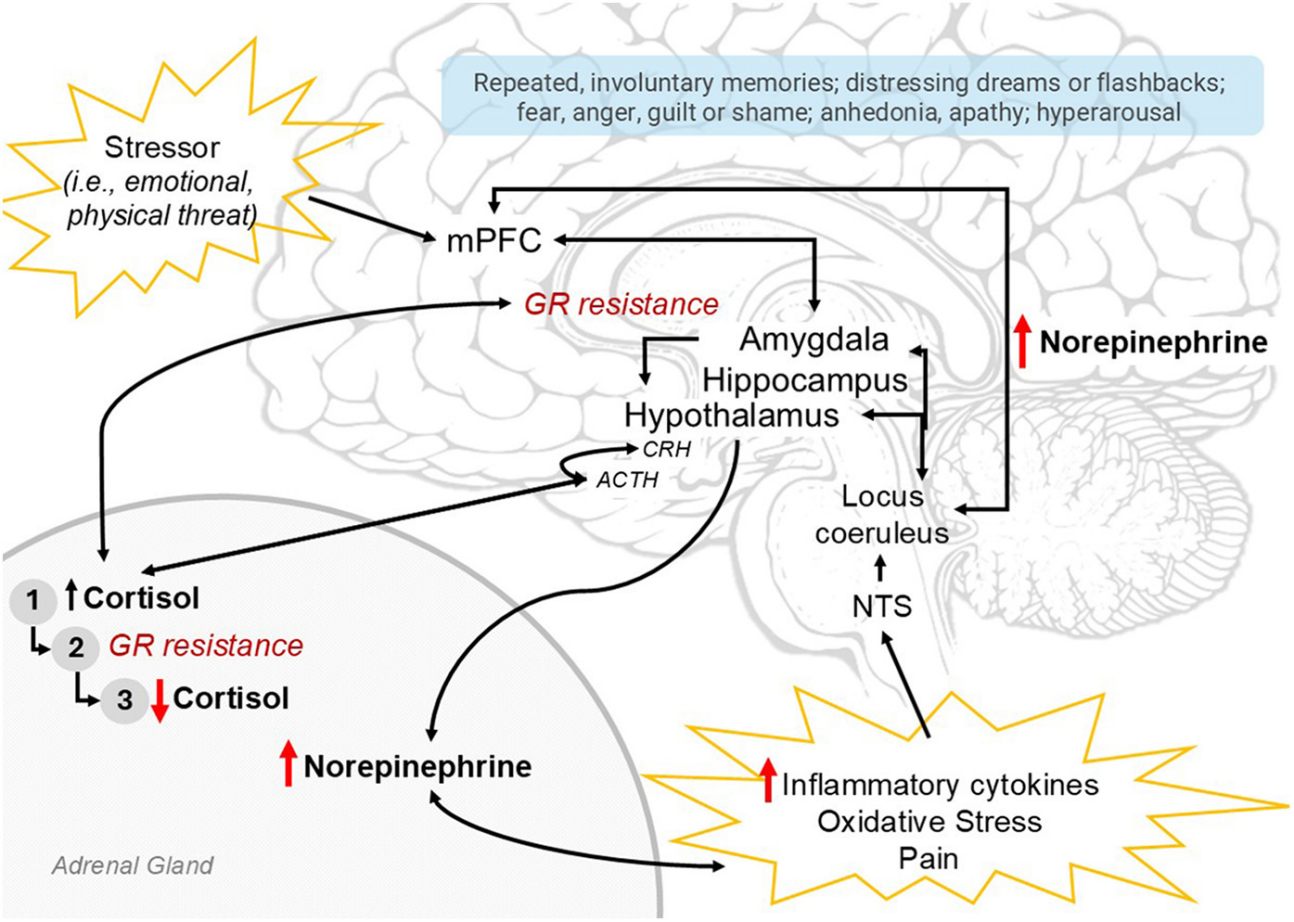
Summary of neuroendocrine profile of PTSD pathophysiology. The stress responses and endophenotype of PTSD are depicted here. The stress response may be activated by a perceived stressor (emotional, physical threat; top left) appraised by the mPFC, amygdala and hippocampus, which may result in hypothalamic activation and initiation of the sympathetic nervous system (part of which involves norepinephrine release from the adrenal medulla) and the hypothalamic-pituitary-adrenal (HPA) axis (CRH → ACTH → Cortisol). Alternatively, these systems are activated by peripheral norepinephrine signalling in response to inflammatory cytokines, oxidative stress, +/- pain, with ascending signals through the nucleus of the solitary tract (NTS), the locus coerulus, and ultimately widespread throughout the central nervous system. With sustained HPA-axis activation, (1) cortisol initially increases; then, with sustained elevation, induces (2) glucocorticoid-receptor (GR) resistance and loss of HPA-axis inhibition, resulting in further increasing cortisol levels until finally the adrenals are depleted resulting in (3) low cortisol. Elevated norepinephrine and low cortisol perpetuate peripheral inflammation and pain, resulting in continued stress response; central norepinephrine elevation contribute to symptoms of hyperarousal and anxiety. Ultimately, the neurophenotype of PTSD (red arrows) involves HPA-axis dysregulation, low cortisol, high norepinephrine and inflammation. Relevant PTSD symptoms in light blue box. PTSD, posttraumatic stress disorder; mPFC, medial prefrontal cortex; CRH, corticotropin-releasing hormone; ACTH, adrenocorticotropic hormone; GR, glucocorticoid receptor.

In response to a threat, as appraised by either the central nervous system (CNS) or by peripheral autonomic signalling, the stress response activates the sympathetic nervous system (SNS) and the hypothalamic-pituitary-adrenal (HPA) axis (Figure 2). Ideally, the stress response leads to activation of adaptive systems to address a physiological challenge (13). However, in extreme cases such as during critical illness, chronic or exaggerated stress responses can disrupt the system which becomes maladaptive. For a comprehensive review of stress circuity and pediatric PTSD pathophysiology, see Pervanidou et al. (3).

CNS stress appraisal involves neural circuitry coordinating fear learning, memory consolidation, and neuroendocrine signalling to systematically coordinate a response. Specifically, this involves the frontal lobe and executive centers, particularly the medial prefrontal cortex (mPFC); the amygdala or “fear” center which processes aversive input with context from the hippocampus, a primary site for memory consolidation; and the hypothalamus, which centrally coordinates neuroendocrine responses (Figure 2) (3). The mPFC may inhibit the amygdala if a stressor is determined as non-threatening. In PTSD, the suspected neurophenotype involves mPFC and hippocampal atrophy with amygdala hypertrophy (14), reflecting a hyperactive fear response and loss of executive inhibition. Clinically, symptoms involve hyperarousal, hypervigilance, intrusive memories/nightmares, anxiety and fear.

Homeostatic systems, including the stress response, are regulated through negative feedback. Cortisol, the end-effector of HPA-axis activation, is sensed by glucocorticoid receptors (GRs) in the amygdala, hippocampus, and hypothalamus, inhibiting further HPA axis activation (and to a degree, SNS activation and NE release; Figure 2) (3). High exposure to cortisol over time results in downregulation of GRs and induced resistance to activation. This impairs negative feedback and potentiates ongoing HPA axis activation, ultimately depleting adrenal stores resulting in relative adrenal insufficiency and hypocortisolemia (3) a phenomenon long recognized in critical care (15). Chronic, unpredictable, or uncontrolled stressors produce an exaggerated stress response (5), and HPA axis dysregulation. Aberrant HPA-axis function also elevates norepinephrine (NE) signalling, increases inflammation from GR-resistance in immune cells, and increases pain (Figure 2), all of which further activate the stress response through peripheral ascending signals and likely PTSD (13, 16). Ultimately, the suspected endophenotype of pediatric PSTD involves a dysregulated HPA-axis, GR-resistance, and elevated NE.

### 2.2 Steroids and PICU-PTSD

Specific to PICU-PTSD, HPA-axis dysfunction in PICU-PTSD is suggested in observational studies that showed abnormally high cortisol associated with PTSD in 47 child PICU survivors (17) and higher severity of PTSD symptoms with decreasing cortisol at 2-weeks and 3-months post PICU discharge (18). Unexpected or emergency PICU admissions, higher severity of illness, and invasive procedures are associated with greater PICU-PTSD (2). Interestingly, in many adult PTSD studies and one in PICU survivors, a single dose of glucocorticoid after a stressor facilitated memory extinction and reduced the probability of PICU-PTSD (19). The effect of cortisol on memory consolidation is dose-dependent (5), and higher cortisol levels augmented by exogenous administration may impair trauma-memory related consolidation. The timing and dose of steroid administration required is likely very nuanced, and currently little is known to make any clinical recommendation. This complexity may explain why a more recent study found no difference in PTSD in 24 out of 65 children who received steroids during their course of septic shock vs. those who did not (20). In that study, steroid administration was described generally, with no specifics on timing and dose. As a potential protective factor in PICU-PTSD, this is an important avenue for future research and rigorous prospective study.

### 2.3 Norepinephrine (NE)

NE is a key neurotransmitter in autonomic and endocrine regulation; learning, memory and plasticity; attention, arousal, and pain evaluation (Figure 2) (14). Patients with PTSD are demonstrated to have higher basal levels of NE, greater NE responsiveness, and higher cerebrospinal fluid NE levels correlated to increased PTSD symptom severity (13). Adolescents with PTSD have blunted cardiac output reactivity and increased vascular resistance in response to stress (3). In PTSD, NE is suspected to be elevated due to process that drive a hyperactive SNS and/or decreased vagal activity (3, 13), such as loss of NE inhibition from GR-resistance and decreased number and efficacy of *α*-2 and increase in NE transmission from amygdala hyperactivity and decreased NE clearance (13). NE affects the impact of glucocorticoids on memory consolidation in a dose-dependent manner, with higher levels of NE disrupting attention and working memory and possibly disinhibiting amygdala fear responses, likely contributing to intrusive thoughts, flashbacks, and repetitive nightmares seen in PTSD (5). While NE in adult and pediatric PTSD is well studied, there are currently no studies examining endogenous or exogenous NE related to PICU-PTSD.

Therapeutically, there has been interest in beta-blockers and *α*-2 receptor agonists to address this hyperadrenergic state. While beta-blocker administration immediately after a trauma has shown some efficacy in preventing adult PTSD (3), a large randomized controlled trial comparing the effect of propranolol treatment to none in 197 pediatric burn patients found no difference in PTSD (21). Activation of *α*-2 autoreceptors inhibits norepinephrine release and stimulates fronto-cortical glutamatergic neurons that improves frontal lobe functioning and efficiency, including working memory and amygdala inhibition (13, 22). The American Psychiatric Association lists *α*-2 receptor agonists as an alternative therapy for symptoms of hyperarousal, and while this is gaining recognition, there is currently little evidence to support this clinical practice (22). Dexmedetomidine is a *α*-2 receptor agonist recommended as first line sedation in the most recent international PICU guidelines (23). Only one observational study in PICU patients examined general dexmedetomidine exposure to PTSD, with no association (24). However, given PTSD and *α*-2 receptor agonist pathophysiology and the potential protective effect from a therapy already routinely used in PICU practice, this area warrants future rigorous study.

### 2.4 Glutamate, gamma-aminobutyric acid (GABA), and neuroplasticity

Glutamate is a widespread excitatory neurotransmitter that fundamentally affects emotion, cognition and HPA-axis regulation (25). Decreased glutamatergic tone in the mPFC and hippocampus perpetuate HPA-axis dysregulation (25). During chronic or severe stress, dysregulated glutamate transmission occurs with extra-synaptic glutamate spillover, causing glial deficits and decreased glutamate signalling due to an upregulation in autoreceptors. This leads to a paradoxical increase in extrasynaptic glutamate and resulting excitotoxicity, driving synaptic dysconnectivity and ultimately PTSD symptomatology through fear and emotional dysregulation (26).

PICU care sometimes involves administration of glutamate-modifying medications, including ketamine. Through preferential binding to the glutamate N-methyl-D-aspartate (NMDA) receptor located on inhibitory GABA interneurons, ketamine produces an uncoordinated increase in excitatory neural activity. Ketamine primarily affects the cortex, amygdala, and hippocampus, the main neurocircuitry important to PTSD (27), increases brain-derived neurotrophic factor (BDNF) promoting synaptogenesis, and blocks stress-related memory impairment (28). A recent meta-analysis in adult PTSD patients found one intravenous dose of ketamine (0.5 mg/kg to 1 mg/kg) was associated with symptom improvement 24 hours after the first infusion, and at the conclusion of the treatment period with decreased PTSD-related hospitalizations (28). Ketamine treatment did not acutely increase symptoms of PTSD, despite its hallucinogenic properties. In pediatric patients, a meta-analysis of ketamine administration for depression (which has similar neurophysiology and therapeutic target for ketamine as PTSD) showed significant efficacy without severe adverse events (29).

The implication for PICU care is not clear. PTSD treatment with ketamine as described occurred after the diagnosis, not during the traumatic event (such as PICU admission) prior to disease development. Ketamine used during PICU care is associated with delirium, and delirium is associated with PICU-PTSD (23, 30). One observational study identified increased PTSD in PICU survivors related to ketamine exposure (31). Further study is required to better understand the impact of ketamine as either a precipitating or protective factor in PICU-PTSD.

Another commonly used PICU medication that modulates GABA transmission, benzodiazepines, has been associated with increased PICU-PTSD (20, 24). In adults, a meta-analysis demonstrated benzodiazepine administration shortly after a traumatic event makes an individual 2–5 times more likely to develop subsequent PTSD (32). Benzodiazepines agonize GABA-A receptors, globally depressing executive function (including the mPFC) which impacts threat appraisal, cognitive and emotional processing and disinhibits fear responses (32), with markedly less activity and therefore depression of amygdala and hippocampal function. This mimics the neurocircuitry implicated in PTSD, of a hyperresponsive amygdala and decreased mPFC inhibition. This effect, in addition to the association of benzodiazepines with delirium (23), likely contributes to PTSD pathology. Post-PICU, benzodiazepine use likely perpetuates PTSD. Despite their use in anxiety disorders, benzodiazepines are ineffective in PTSD treatment and even worsen prognosis.

### 2.5 Inflammation

PTSD is associated with immune dysfunction (16). Hyperadrenergic stimulation of immune cells and reduced cortisol immunomodulation from GR-resistance promote a pro-inflammatory state. Inflammation itself triggers SNS-activation through visceral signalling, further perpetuating the cycle. Epigenetic changes observed in PTSD relate to inflammatory-regulator genes, and increased inflammation pre-trauma exposure increased risk for subsequent PTSD (16). Neuroinflammation contributes to neuropsychiatric disorders and symptoms (16, 33) including fatigue, anhedonia, impaired concentration, and impaired fear memories. In PICU-PTSD, observational studies have shown sepsis was independently associated with PTSD (17, 34). Within septic patients, higher levels of inflammatory markers (C-reactive protein) were independently associated with increased PTSD scores (20, 35). Sepsis, as a clinical syndrome, is also associated with endothelial dysfunction and altered lactate metabolism. Endothelial dysfunction is hypothesized to contribute to PTSD vulnerability by increasing blood-brain-barrier permeability and stress-signalling to neurological networks (36). The potential function of lactate in cellular signalling and epigenetic changes in PTSD is an emerging area of study with relevance to sepsis (37). Future studies may explore personalized therapies to modulate hyperinflammation as a potential therapeutic approach.

### 2.6 Mechanical ventilation

Brain-lung bidirectional interactions are complex and well described in critical care research; see an excellent 2021 overview by Albaiceta et al. (38) Both acute and chronic brain dysfunction are described secondary to mechanical ventilation (MV) (39). Presumed mechanisms include MV-associated systemic inflammation and autonomic dysfunction resulting in neuroinflammation (including within the amygdala), and hippocampal cell death (38). In a scoping review of ventilated adult acute respiratory distress syndrome patients, secondary acute brain injury or poor neurological outcome was reported as high as 82%–86% (40). A propensity-matched review of a medical data repository identified that in 1,351 mechanically ventilated children, there was a 43% higher incidence of subsequent mental health diagnoses, and a 67% higher incidence of psychotropic medication use compared to 6,755 matched general inpatients (41). Interestingly, although the authors found a higher risk of mental health diagnoses overall, they did not find increased risk of PICU-PTSD. This may reflect an underdiagnosis of PTSD (42) as they measured retrospective diagnostic codes. Further studies are required to better understand the relationship between respiratory disease and neurological outcomes, particularly as brain-protective ventilation is a potentially modifiable PICU variable.

## 3 Psychological factors

Cognitive models of PTSD describe high levels of dissociative reactions, fear, and maladaptive processing during a traumatic event with subsequent rumination and avoidance (43). Specific psychological aspects related to PICU-PTSD risk include delirium, patient cognition and psychological factors, and parental anxiety with PTSD.

### 3.1 Delirium and cognitive processing

Delirium, the most common cause of acute brain dysfunction in PICU, is associated with inflammation and neuronal apoptosis that may lead to brain atrophy in the PFC and hippocampus (19). Delirium pathophysiology in part, relates to arousal and attention related to aberrant norepinephrine signalling (44) and impacts the quality of cognitive processing, which has been associated with subsequent PICU-PTSD (31). More awake adult patients during MV had the lowest PTSD symptoms (19). Delirium, delusional memories, and delirium-precipitating medications (midazolam and opioids) are independently associated with PICU-PTSD in observational studies (24, 30). ICU-diaries, a low-cost and low-harm intervention, improves PTSD symptoms in patient survivors and families in adult randomized trials (19), possibly by improving cognitive processing.

### 3.2 Patient survivor psychological characteristics

PICU survivors’ cognitive and emotional characteristics, specifically peri-trauma affect, cognitive processing, anxiety, and trauma memory, have been associated with PICU-PTSD (31, 45–47). Other psychiatric diagnoses, such as anxiety and major depressive disorder, are often comorbid with PTSD (48) with suspected common underlying pathophysiology such as neuroinflammation, HPA-axis and norepinephrine dysregulation (14). This may, in part, play a role in why pre-existing psychiatric problems are associated with increased risk of PICU-PTSD (47) and co-morbid anxiety mediated the progression of acute stress disorder to PICU-PTSD in adolescents (49). Maybe unsurprisingly, PTSD at 3-months post-PICU was associated with PTSD diagnosis at a later follow-up in several observational studies (7), possibly reflecting a lack of treatment or chronic PTSD trajectory post-PICU (47). All of these data emphasize the importance of recognition and treatment of PICU-PTSD and other psychiatric diagnoses.

### 3.3 Parental anxiety and PTSD

Parental PTSD has been strongly associated with survivor PTSD in many observational studies (2), albeit noting that many PTSD screening assessments are parental reports which may overestimate prevalence (47). Associations between parental and child PTSD is likely multifactorial. There is a strong genetic basis for PTSD with 24%–72% of PTSD risk inherited (16). Different genetic glucocorticoid endophenotypes such as the FKBP5 polymorphism were identified in women and adolescents and predispose the stress system to dysregulation and PTSD (13). In-utero exposure of the fetus to maternal stress, depression, or PTSD, may precipitate epigenetic changes in the fetus that increase methylation of key steroid and inflammatory regulator genes (50). Parents with PTSD may have lower parental care which increases DNA methylation in offspring (50), and likely interferes with a parents’ ability to cope, appraise and respond to child distress. Parental maladaptive coping and perception of illness severity impacts the duration of PTSD symptoms and increases PICU-PTSD in child survivors (43, 51), and conversely, an intervention aimed to help support, educate and empower mothers decreased PICU-PTSD in survivors (52). Altogether, this interplay in genetics, epigenetics, psychological coping and care contributes to intergenerational trauma (53). Emerging evidence demonstrates improved psychosocial support and empowerment provided to families by the PICU during hospitalization, combined with psychoeducation post-discharge, improves psychological symptoms and PTSD in parents and children (4). There is an opportunity here for PICU to better support caregivers, and by extension, prevent PTSD in the entire family, warranting further consideration and research.

## 4 Social factors

A key component of the biopsychosocial model of disease is acknowledging the importance of social context and the impact on the individual (54). In PTSD, everything from traumatic exposure type and risk to systemic support and vulnerabilities are influenced by social context (55). Despite the importance of social factors on the pathophysiology of PTSD, a limited number have been directly studied so far, representing an important area for future study.

### 4.1 Family presence in PICU and social support

The concept of personhood and person-centered care includes social relationships with healthcare providers, communication, comfort, attachment and identity. A qualitative study in adults emphasized the importance of person-centered case in ICU in patient self-determination and health outcomes (56). Within pediatrics, data on person-centered care related to PICU-PTSD is limited. Often, the focus is on family-centered care with the child seen in the context of the family unit. Family member presence in PICU was protective against the development of PICU-PTSD in one study (57), consistent with numerous studies demonstrating poor mental health consequences in on patients, families and caregivers who experienced restricted family presence during the coronavirus pandemic (58). Low social support has been associated with heightened stress reactivity, specifically increased noradrenergic signalling and HPA-axis reactivity (59). Further research is required to better understand this potential relationship in PICU-PTSD. Acknowledging resources and support available to families on a case-by-case basis is an important aspect of PICU care, as is the importance of facilitating family presence at the bedside. PICUs may consider addressing barriers and enablers to family presence in the PICU, well described by Poole et al. (60) in semi-structured interviews with 14 primary caregivers.

### 4.2 Equity and adverse childhood experiences

There are systemic disparities in the social systems related to repeated exposure and the type of traumatic events or adverse experiences during childhood (61). There is an increased probability and greater severity of lifetime PTSD among racial, ethnic and sexual minorities and low-income populations (62, 63). It is very important to recognize system factors and avoid attribution bias by incorrectly assuming biological differences increase the risk of PTSD, rather than through the contact of the social system. For example, women are twice as likely to develop PTSD even though men report higher frequency of exposure to traumatic events. While there are neuroendocrine differences between males and females, this is likely due to disproportionate burden of sexual violence and assault experienced by women, which is associated with the highest rates of PTSD (64).

Within PICU, disparities exist due to race or ethnicity and socioeconomic position in management and outcomes of asthma, severe trauma, sepsis, oncology, out-of-hospital cardiac arrest, and families’ perception of care (65). There is a paucity of related PICU-PTSD data, despite well-studied disparities in adult and non-medical PTSD. One case-control study by Als et al. (17) looked at “vulnerability factors” in 47 children, finding ethnicity and past health problems were associated with PTS symptoms. A recent secondary analysis of a large randomized multi-center trial evaluating sedation titration for respiratory failure found lower median income was significantly associated with elevated PTSD at follow-up (66). Challenges in this sphere of research include heterogenous measures of socioeconomic status making a future pooled analyses problematic, and under-enrollment of participants from minority groups (65). Future research on social system inequities related to PICU-PTSD will be very important to recognize and address social constructs and context that impacts patients (67).

## 5 Conclusion and future directions

PICU-PTSD is a functional, structural, pharmacological and biochemical disorder that greatly affects children and families after PICU admission. HPA-axis dysregulation and GR-resistance, altered ANS balance, glutamate and GABA dysfunction, inflammation, cognitive processing and psychosocial support are important processes in PTSD pathophysiology that directly relate to PICU care. While largely observational, a large body of work identifying contributors to PICU-PTSD may be applied to a biopsychosocial framework to better understand the disorder and work towards improved identification, prevention and treatment for families. Currently, there is a paucity of data on effective interventions for PICU-PTSD (68). One study examined propranolol treatment during PICU admission to prevent PTSD and found no effect (21). Another interventional randomized controlled trial found implementing a preventive educational-behavioral intervention program reduced PICU-PTSD at follow-up (52). Future interventional studies examining potentially modifying therapies to prevent PICU-PTSD may examine factors such as steroid administration, sedation and analgesia practices, delirium prevention and treatment, parental and patient support with encouragement of family presence at the bedside and improving mental health literacy. Until then, units could consider implementing a risk-assessment bundle for PICU-PTSD using this biopsychosocial framework to identify and triage at-risk patients for better recognition and care in follow-up. Factors requiring further study and not described here, include environmental factors such as the impact of climate change (21). Similarly, development of further PICU-PTSD frameworks may consider macrocosmic elements such as climate change, technology, and larger societal structures, perhaps through a general systems theory approach.
